# Radiomic analysis based on magnetic resonance imaging for the prediction of VEGF expression in hepatocellular carcinoma patients

**DOI:** 10.1007/s00261-024-04427-0

**Published:** 2024-06-19

**Authors:** Cui Yang, Ze-Ming Zhang, Zhang-Ping Zhao, Zhi-Qing Wang, Jing Zheng, Hua-jing Xiao, Hong Xu, Hui Liu, Lin Yang

**Affiliations:** 1 Department of Radiology, Panzhihua Central Hospital, Panzhihua, 617000 Sichuan China; 2 Medical Imaging Key Laboratory of Sichuan Province, Science and Technology Innovation Center, Interventional Medical Center, Department of Radiology, Affiliated Hospital of North Sichuan Medical College, Nanchong, 637000 Sichuan P. R. China; 3 Department of Pathology, Panzhihua Central Hospital, Panzhihua, 617000 Sichuan China

**Keywords:** Hepatocellular carcinoma (HCC), Targeted therapy, Magnetic resonance imaging (MRI), Radiomics, Vascular endothelial growth factor (VEGF), Angiogenesis

## Abstract

**Objective:**

The purpose of this study was to investigate the ability of radiomic characteristics of magnetic resonance images to predict vascular endothelial growth factor (VEGF) expression in hepatocellular carcinoma (HCC) patients.

**Methods:**

One hundred and twenty-four patients with HCC who underwent fat-suppressed T2-weighted imaging (FS-T2WI) and dynamic contrast-enhanced magnetic resonance imaging (DCE-MRI) one week before surgical resection were enrolled in this retrospective study. Immunohistochemical analysis was used to evaluate the expression level of VEGF. Radiomic features were extracted from the axial FS-T2WI, DCE-MRI (arterial phase and portal venous phase) images of axial MRI. Least absolute shrinkage and selection operator (LASSO) and stepwise regression analyses were performed to select the best radiomic features. Multivariate logistic regression models were constructed and validated using tenfold cross-validation. Receiver operating characteristic (ROC) curve analysis, calibration curve analysis and decision curve analysis (DCA) were employed to evaluate these models.

**Results:**

Our results show that there were 94 patients with high VEGF expression and 30 patients with low VEGF expression among the 124 HCC patients. The FS-T2WI, DCE-MRI and combined MRI radiomics models had AUCs of 0.8713, 0.7819, and 0.9191, respectively. There was no significant difference in the AUC between the FS-T2WI radiomics model and the DCE-MRI radiomics model (*p* > 0.05), but the AUC for the combined model was significantly greater than the AUCs for the other two models (*p* < 0.05) according to the DeLong test. The combined model had the greatest net benefit according to the DCA results.

**Conclusion:**

The radiomic model based on multisequence MR images has the potential to predict VEGF expression in HCC patients. The combined model showed the best performance.

**Graphical Abstract:**

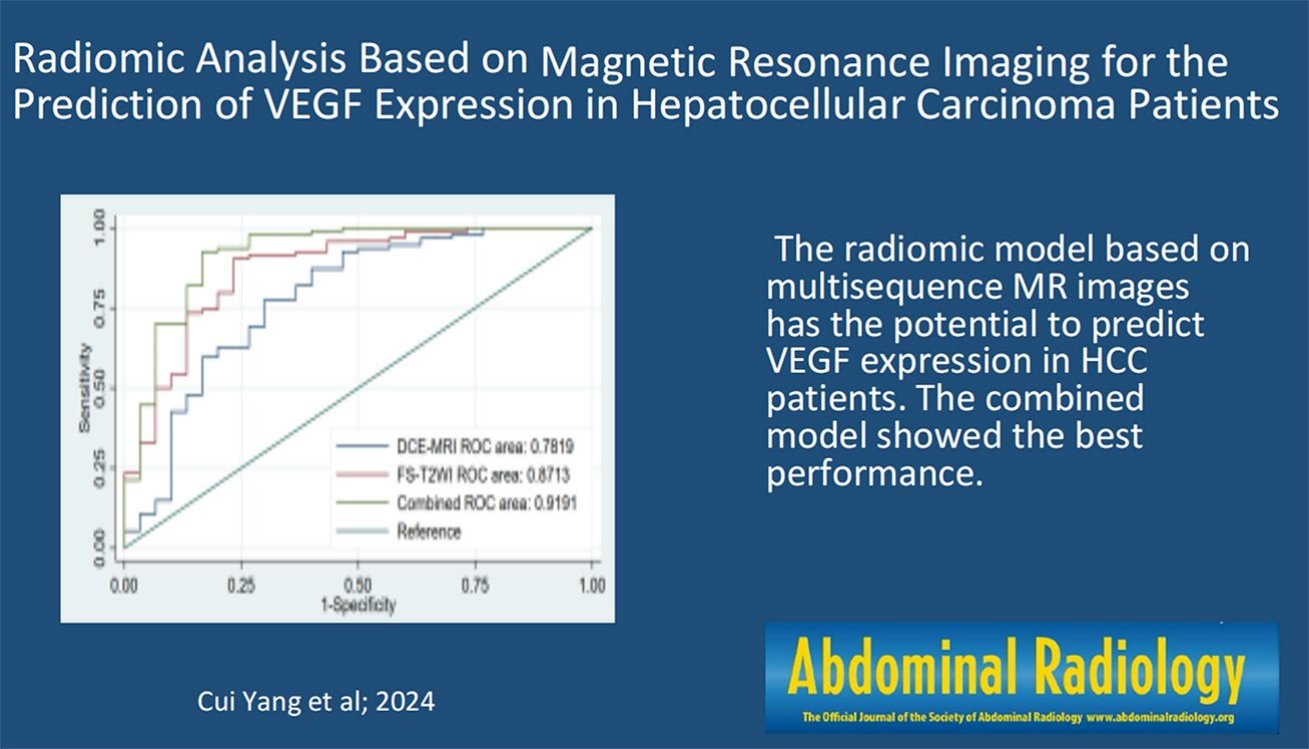

## Introduction

Primary liver cancer ranks fifth in terms of global incidence, and hepatocellular carcinoma (HCC) accounts for 75–85% of liver cancer cases [1]. Most patients with HCC are diagnosed in the middle or advanced stages and miss the opportunity to receive radical surgical resection. Fortunately, systemic therapy has evolved into molecular targeted therapy and immunotherapy. Benefiting from combination treatment involving immune checkpoint inhibitors (ICIs) combined with an antivascular endothelial growth factor (anti-VEGF) monoclonal antibody, advanced HCC is considered highly treatable [2]. Suppressing tumor angiogenesis inhibits tumor growth [3]. As VEGF is a major driver of tumor angiogenesis, efforts have focused on novel therapeutics aimed at inhibiting VEGF activity, and antiangiogenic therapies have shown efficacy in treating HCC [3–5]. Differences in VEGF expression levels may affect the therapeutic effect [6]. Therefore, preoperative noninvasively predicting VEGF expression could be helpful for formulating personalized treatments and predicting the prognosis of HCC patients. In recent years, a new concept of radiomics, automatic high-throughput extraction of many image features based on image analysis, has been proposed [7]. Magnetic resonance imaging (MRI)-based radiomics has been reported in many clinical areas [8], including angiopoietin‑2 (Ang-2) expression, PD-L2 expression, PD‑1/PD‑L1 expression in HCC [9,10,11]. However, few studies have addressed the value of radiomic models based on multisequence MRI in the noninvasive prediction of VEGF expression in HCC patients [6]. Therefore, this study investigated the accuracy of noninvasive prediction of VEGF expression based on MRI radiomics in patients with HCC.

## Materials and methods

### Patient data

One hundred and twenty-four patients with HCC who underwent surgical resection and had no other liver disease according to the inclusion and exclusion criteria were enrolled. The inclusion criteria were as follows: (1) received surgery within 1 week after MRI examination; (2) had not received preoperative radiochemotherapy or preoperative interventional therapy; and (3) had a postoperative pathological diagnosis of HCC. The exclusion criteria were as follows: (1) contraindications for MRI examination; (2) poor image quality; and (3) maximum diameter of the liver lesion < 10 mm.

### MRI scan and data measurement

All patients underwent fat-suppressed T2-weighted imaging (FS-T2WI) and dynamic contrast-enhanced MR using a 3.0 T magnetic resonance scanner with a 32-channel phased array body coil (Discovery MR750, GE, USA). Patients fasted for more than 4 h and completed respiration training before the MRI scans. Each patient was scanned from the apex of the diaphragm to the lower edge of the liver. The MRI sequence and parameters and the data measurement method used in this study were the same as those used in previous studies [9,12]. The sequences were as follows: breath-hold transverse-axis fat-suppressed T1-weighted imaging (T1WI), breath-triggered transverse-axis fat-suppressed T2WI, and dynamic contrast-enhanced (DCE) MRI. The T1WI sequence parameters were as follows: repetition time (TR)/echo time (TE), 4 ms/2 ms; fractional anisotropy (FA), 12; matrix, 260 × 192; field of view (FOV), 36 cm×36–40 cm×40 cm; and slice thickness/interslice gap, 5 mm/0 mm. The T2WI sequence parameters were as follows: TR/TE, 2609 ms/97 ms; FA, 110.0; matrix, 384 × 384; FOV, 36 cm×36–40 cm×40 cm; and slice thickness/inter- slice gap, 5 mm/1 mm. For multiphase DCE-MRI, the contrast agent (Gd-DTPA, 15–20 ml) was injected with a high-pressure syringe through the vein of the hand at an injection speed of 2-2.5 ml/s. Then, arterial and portal phase images were collected.

### Immunohistochemical staining

The VEGF antibody was obtained from Abcam (UK) at a dilution of 1:200. Postoperative HCC tissue specimens were fixed in 10% formalin, dehydrated in ethanol, cleared in xylene, embedded in paraffin, and serially sectioned into 5-µm slices for immunohistochemical staining. The VEGF staining results were evaluated based on the semiquantitative method of Shimizu et al [13].

**Fig. 1:**
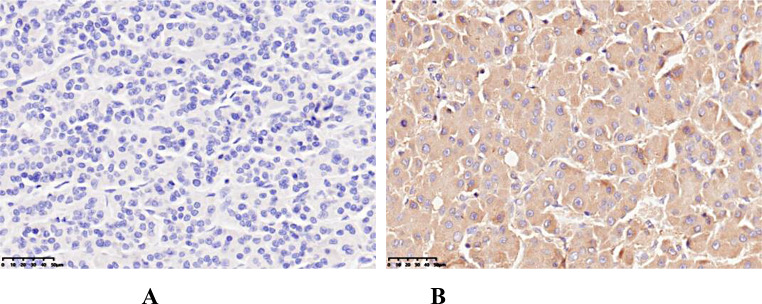
Immunohistochemical staining of HCC tissue using a VEGF antibody (**A**) low VEGF expression (× 200); (**B**) high VEGF expression (× 200)

The scores for the proportion of stained cells were as follows: 0 points indicated that no cells were stained, 1 point indicated that 1–10% of tumor cells stained positively, 2 points indicated that 11–50% of tumor cells stained positively, and 3 points indicated that 51–100% of tumor cells stained positively. Scores for staining intensity were as follows: 0 points indicated that no cells were stained, 1 point indicated that the tumor cells were stained light yellow, 2 points indicated that the tumor cells were stained brownish yellow, and 3 points indicated that the tumor cells were stained brown. The overall score was defined as the sum of the scores for the proportion of stained cells and for staining intensity: overall score = score for the proportion of stained cells + score for staining intensity. The overall score was divided into two levels: low VEGF expression (< 3 points) and high VEGF expression (≥ 3 points). For controversial results, the final score was reached through discussion by two pathologists (Fig. 1).

### Tumor segmentation and feature extraction

**Fig. 2:**
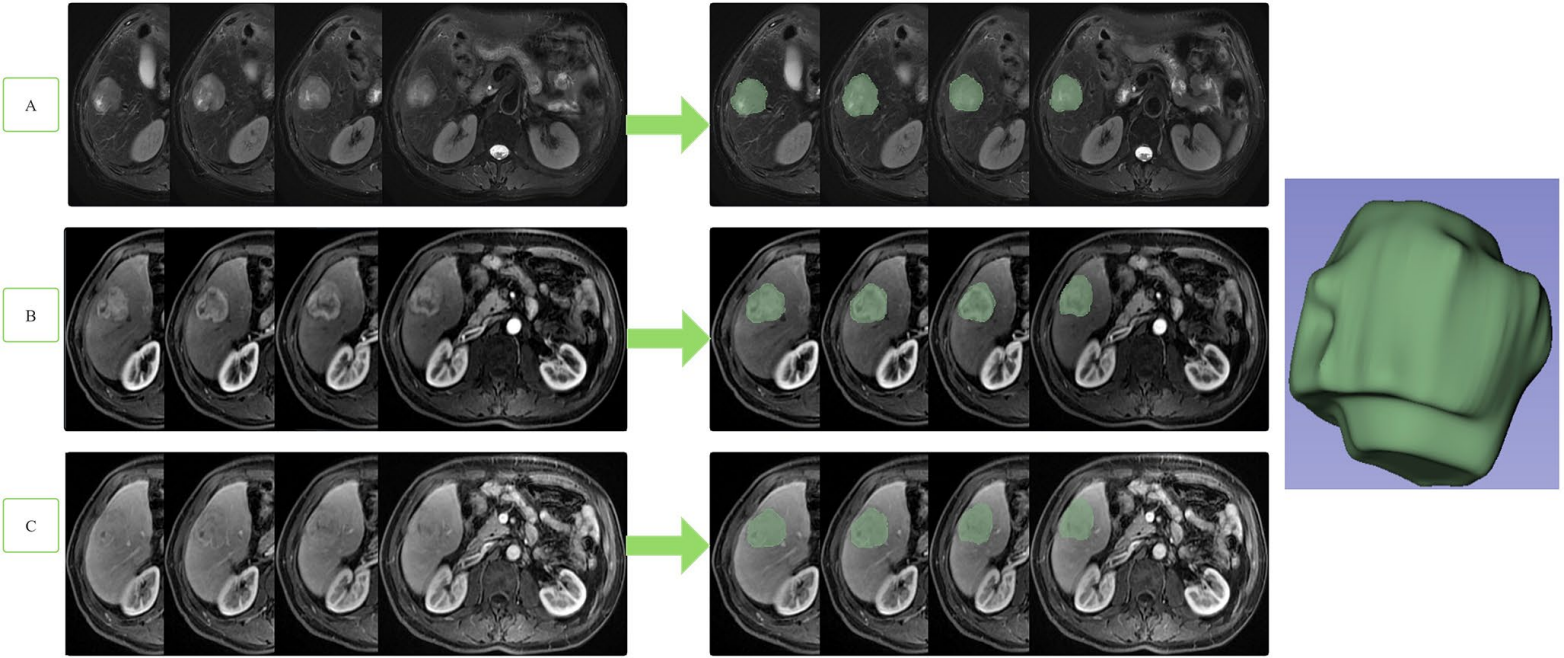
The volume of interest (VOI) was delineated layer by layer on axial FS-T2WI (**A**), in the arterial phase (**B**), in the portal venous phase (**C**), covering the entire tumor and avoiding areas of surrounding bile ducts and blood vessels as much as possible

Images of the study objects were downloaded and exported through the picture archiving and communication system (PACS) and imported into 3D-Slicer software [14–16]. Two radiologists (observer 1 and observer 2) with 6 years of experience in abdominal MR diagnosis manually delineated the volume of interest (VOI) layer by layer on axial FS-T2WI and DCE-MRI (arterial phase and portal venous phase) of axial MRI, covering the entire tumor and avoiding areas of surrounding bile ducts and blood vessels as much as possible (Fig. 2). The derived images were obtained from the original images and passed through the Laplacian of Gaussian filter and the wavelet filter [16]. A total of 1130 features were extracted from the original images and the derived images, including shape-based, texture-based, histogram-based and wavelet-based features. The datasets from FS-T2WI, DCE-MRI (arterial phase and portal venous phase) and the combined feature set of the former two were established. Two observers randomly selected 50 HCC patients to delineate the VOIs on axial FS-T2WI and the arterial phase and portal venous phase of axial MR images. Interobserver agreement was assessed by the intergroup correlation coefficient (ICC). When the ICC was ≥ 0.75, the two observers were in good agreement [17].

## Feature screening and model establishment

Features with ICC ≥ 0.75 were selected based on the consistency test. The missing values in the features were filtered, and the feature data were standardized to ensure the reproducibility of the results. Least absolute shrinkage and selection operator (LASSO) and stepwise regression analysis were used to reduce the dimensionality of the features and obtain the optimal feature set to establish the FS-T2WI model, DCE-MRI model and combined model. The performance of the models was verified by 10-fold cross-validation. The predictive performance of the three models was evaluated by receiver operating characteristic (ROC) curves. To evaluate the models, the area under the curve (AUC) was computed, and calibration curve analysis and decision curve analysis (DCA) were used.

## Statistical analysis

The statistical software R (version 3.6.0) was used for statistical analysis. The independent samples t test or Kruskal‒Wallis nonparametric rank-sum test was used to analyze continuous variables, and the Chi‒square test or Fisher’s exact test was used to compare categorical variables. A two-tailed P value < 0.05 was considered indicative of statistical significance.

## Results

**Fig. 3:**
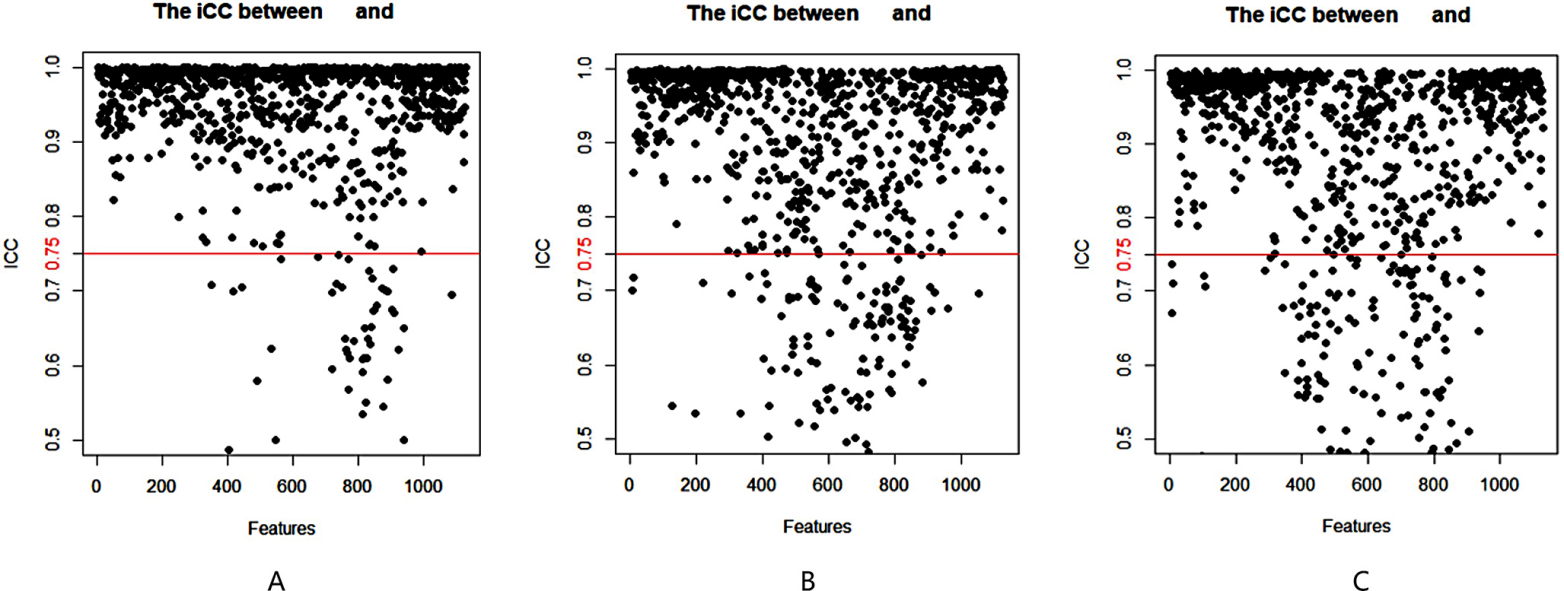
Stability assessment of the extracted MRI radiomics features by the ICC (**A**) FS-T2WI; (**B**) DCE-MRI arterial phase; (**C**) DCE-MRI portal venous phase

**Fig. 4:**
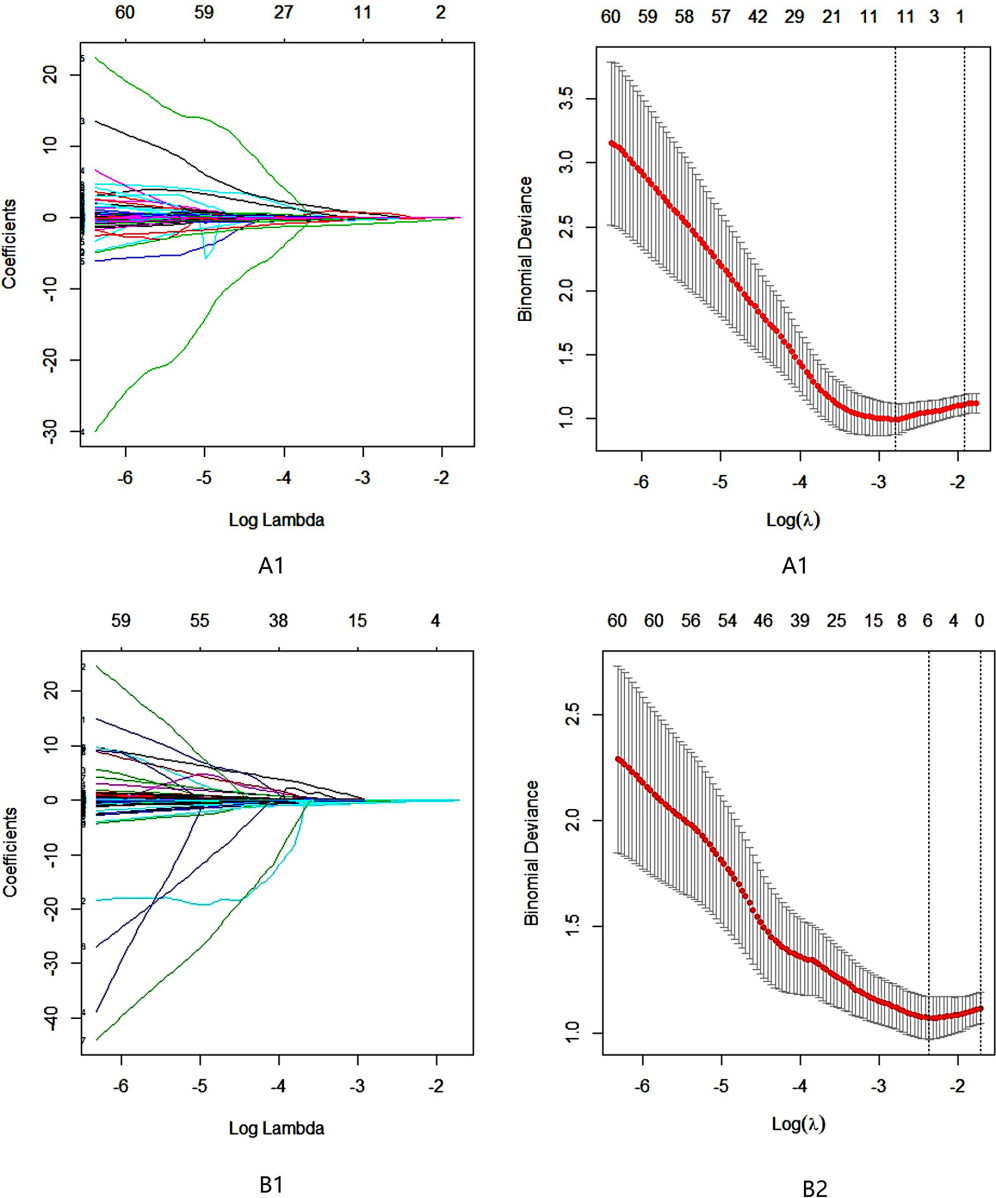
Radiomic feature selection using LASSO regression analysis (A1–A2) FS-T2WI; (B1–B2) DCE-MRI (arterial phase and portal venous phase)

**Fig. 5:**
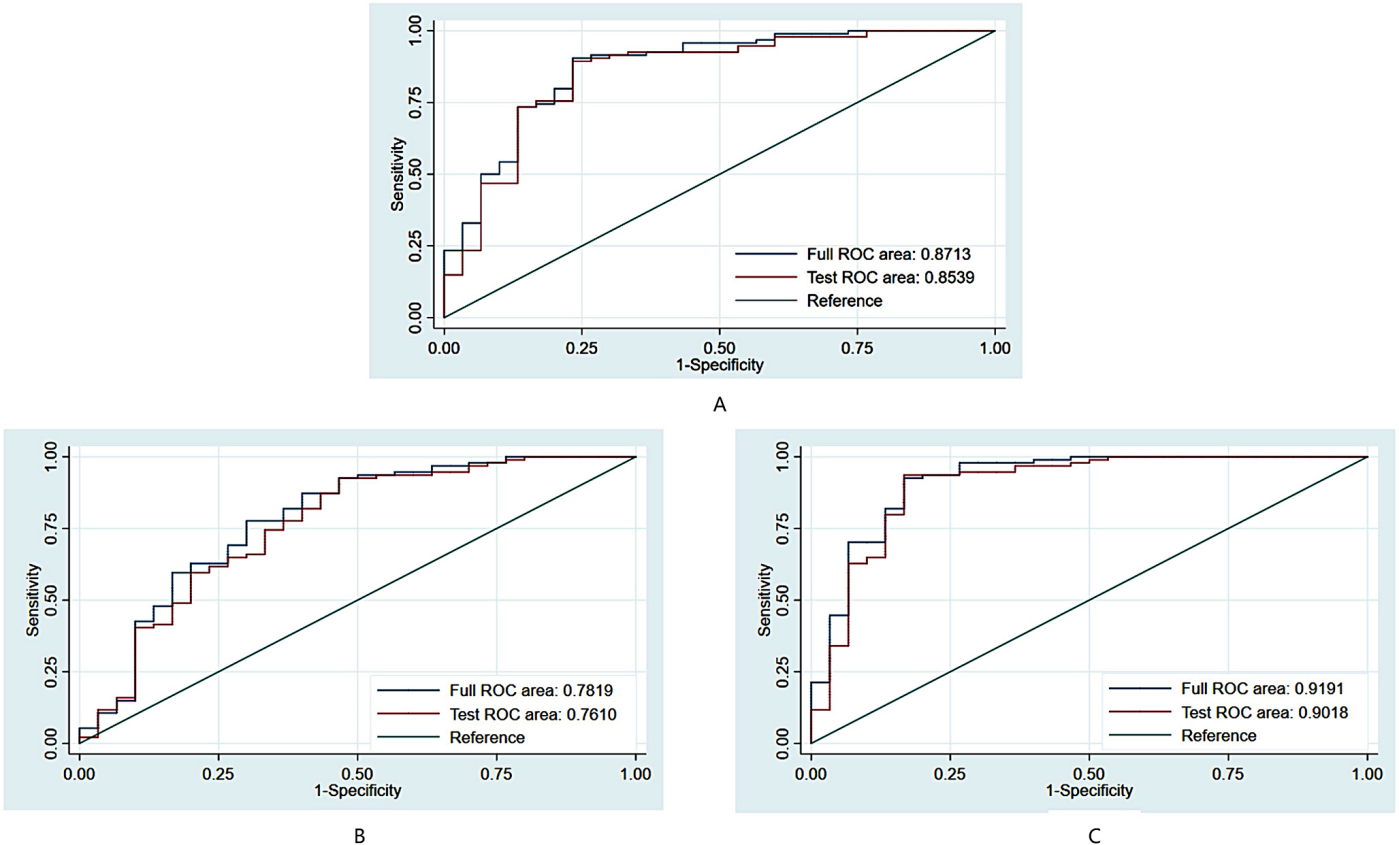
(**A**) ROC curves for the 10-fold cross-validation of the FS-T2WI model; (**B**) ROC curves for the 10-fold cross-validation of the DCE-MRI (arterial phase and portal venous phase) model; (**C**) ROC curves for the 10-fold cross-validation of the combined model

**Fig. 6:**
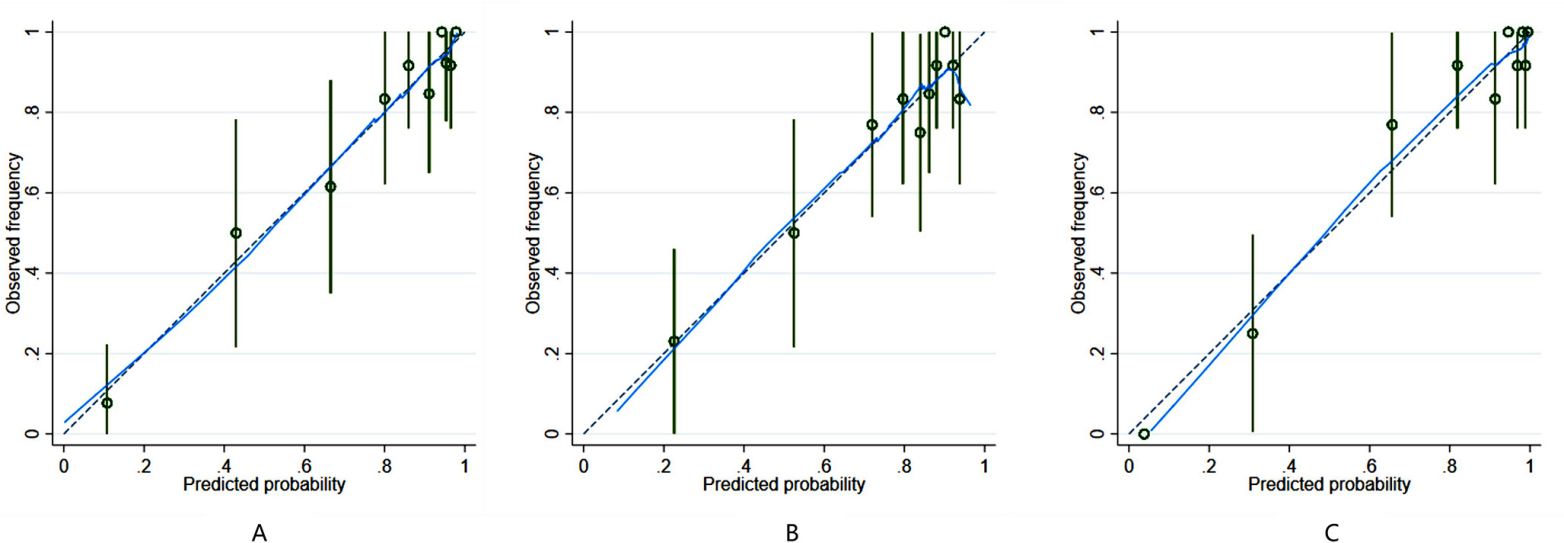
(**A**) FFS-T2WI model calibration curve; (**B**) DCE-MRI model calibration curve; (**C**) combined model calibration curve; calibration curve—the predicted probability of the model and the actual probability; that is, the closer the nomogram is to the ideal curve, the better the ft of the model

**Fig. 7:**
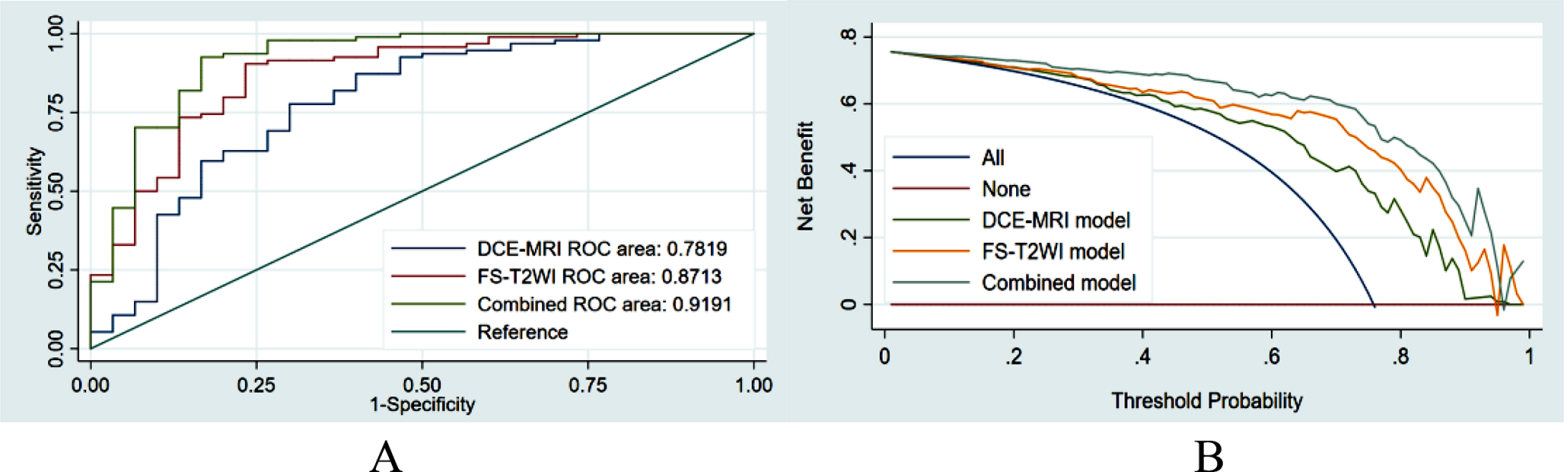
(**A**) comparison of the ROC curves for the prediction of VEGF expression by various models; (**B**) clinical DCA of 3 models; the y-axis represents the standardized net benefit, and the x-axis represents the high risk threshold; dark red (without VEGF expression) and blue (with VEGF expression) represent 2 extreme cases, and it is better if the curve is far from the 2 extreme cases

There were 124 HCC patients in this study, including 110 males and 14 females aged 23 to 73 years, whose longest tumor diameter ranged from 1.5 cm to 16.0 cm; 94 patients had high VEGF expression, and 30 patients had low VEGF expression (Table 1). A total of 1130 image features were extracted from FS-T2W images and arterial phase and portal venous phase images for each patient. After screening, including the intergroup consistency test (Fig. 3), LASSO dimensionality reduction (Fig. 4) and stepwise regression analysis, 5, 3 and 7 optimal features were obtained from the FS-T2WI, DCE-MRI and combined datasets, respectively, to establish the prediction models. The performance of the models was verified by 10-fold cross-validation (Fig. 5). Calibration curves were used to evaluate the models (Fig. 6). The FS-T2WI, DCE-MRI, and combined MRI radiomics models had AUCs of 0.8713 (95% CI: 0.79196–0.95060), 0.7819 (95% CI: 0.67456–0.88927) and 0.9191 (95% CI: 0.85189–0.98640), respectively (Table 2). There was no significant difference in the AUC between the FS-T2WI radiomics model and the DCE-MRI radiomics model (*p* > 0.05), but the AUC for the combined model was significantly greater than AUCS for the other two models (*p* < 0.05) according to the DeLong test. The combined model had the greatest net benefit according to the DCA results (Fig. 7).

**Table 1:** Clinical characteristics of patients in the high and low VEGF expression groups

Clinical Variables	Total (*n* = 124)	High VEGF Expression (*n* = 94)	Low VEGF Expression (*n* = 30)	*p*
Sex				0.092
Male	110	86	24	
Female	14	8	6	
Age (years)				0.022
≤ 60	81	56	25	
> 60	43	38	5	
Diameter (cm)				0.177
0–5	63	51	12	
> 5	61	43	18	
Portal vein tumor thrombus				0.071
No	94	75	19	
Yes	30	19	11	
Liver cirrhosis				0.439
No	27	22	5	
Yes	97	72	25	
Hepatitis B				0.547
No	32	23	9	
Yes	92	71	21	
AFP (ng/mL)				0.799
≤ 400	85	65	20	
> 400	39	29	10	
Ascitic fluid				0.955
No	78	59	19	
Yes	46	35	11	

*NoteAFP*, alpha-fetoprotein

**Table 2:** Predictive performance of each model

Model	AUC (95% CI)	Accuracy	Sensitivity	Specificity
FS-T2WI	0.8713 (0.79196–0.95060)	85.48%	94.68%	56.67%
DCE-MRI	0.7819 (0.67456–0.88927)	82.26%	94.68%	43.33%
Combined	0.9191 (0.85189–0.98640)	91.13%	96.81%	73.33%

*NoteAUC*, area under the ROC curve; CI, confidence interval; FS-T2WI, fat-suppressed T2-weighted imaging; DCE-MRI, arterial phase and portal venous phase; combined, FS-T2WI + DCE-MRI

## Discussion

HCC is considered an angiogenic malignant tumor [18]. Angiogenesis is a hallmark of tumor progression [19], and angiogenesis has been suggested as a potential prognostic factor for poor outcomes in patients with HCC [6]. Tumor angiogenesis is a complex process that is associated with angiogenic factors [19]. Among the known positive regulators, VEGF has emerged as a central regulator of the angiogenic process not only under physiological conditions but also under pathological conditions [20]. High VEGF expression is associated with the development and progression of HCC [21]. VEGF is complex and is a powerful inducer of angiogenesis that stimulates the growth and proliferation of endothelial cells, acts as a survival factor for endothelial cells, prevents the apoptosis of endothelial cells, and regulates vascular permeability [19]. To induce angiogenesis, VEGF interacts with multiple receptors (VEGFR 1, VEGFR 2, VEGFR 3, and neuropilin) [20, 22]. Due to the key role of angiogenesis in HCC development and progression, antiangiogenic therapy for HCC is a highly effective strategy. Molecular targeted drugs have become crucial therapeutic agents for a variety of cancers, and antiangiogenic agents are the only effective molecular targeted therapies for HCC [2]. In 2020, an immune checkpoint inhibitor (ICI) combined with an antivascular endothelial growth factor (anti-VEGF) monoclonal antibody (atezolizumab and bevacizumab) increased the tumor response rate to approximately 30% and overall survival (OS) to approximately 20 months [2]. HCC patients may benefit more from preoperatively predicting VEGF expression.

Solid cancers are spatially and temporally heterogeneous, which limits the use of invasive biopsy-based molecular assays [7]. Radiomics is an approach that holds great promise, as it involves automatic high-throughput extraction of image features from radiographic images and can noninvasively reflect intratumoral heterogeneity [7]. The biological processes occurring at the genetic and molecular levels can be reflected by radiomic features [23]. Radiomic features based on contrast-enhanced T1-weighted and diffusion-weighted images are significantly relevant to the quantitative expression of CD3, CD31, CD68 and PD-L in HCC [24]. Gu et al [25] explored the preoperative predictive value of radiomic features based on contrast-enhanced MR images and identified glypican 3 (GPC3)-positive HCCs, which showed that MR-based radiomic features are closely related to GPC3 expression in HCC. In addition, several studies have investigated the accuracy of predicting Ang-2 expression, PD-L2 expression, and PD‑1/PD‑L1 expression in HCC by preoperative DCE-MRI-based radiomics, T2-weighted-based and DCE-MRI-based radiomics and T2-weighted-based and DCE-MRI-based radiomics, respectively [9–11]. The above three models showed their potential to predict Ang-2 expression, PD-L2 expression, and PD‑1/PD‑L1 expression in HCC patients. These findings may be beneficial for choosing optimal and individualized treatment strategies for HCC patient. The radiomics model based on multiparametric MR features performed best in the training set, consistent with our present study.

Moreover, few studies have explored the value of MRI-based radiomic models in the noninvasive prediction of VEGF expression in HCC patients. Fan et al [6] reported that radiomic features based on contrast-enhanced MR images (from the portal venous and hepatobiliary phases) could be considered credible prognostic markers for the level of VEGF in HCC. The AUC in the training group was 0.892 (95% CI: 0.839–0.945), and the AUC in the validation group was 0.800 (95% CI: 0.682–0.918). However, the researchers only studied contrast-enhanced sequences and did not incorporate other sequences, such as FS-T2WI. MRI sequences are different, the provided information is different, and their contributions to the combined model are also different [26]. Multisequence combined models have been shown to have better prediction efficiency [10, 11, 27–29], consistent with our present study.

In the present study, 5, 3 and 7 optimal features were obtained from the FS-T2WI, DCE-MRI and combined datasets, respectively, to establish the prediction model. Among the optimal features, the wavelet features in all models were the most preserved, consistent with previous studies [10, 30]. Among the 7 optimal features of the combined model, there were six features from FS-T2WI and one feature from DCE-MRI; thus, there were more features from FS-T2WI than from contrast-enhanced sequences, which is inconsistent with the literature [26, 31]. The main reason for this may be that other contrast-enhanced sequences, such as those from the delayed phase and the hepatobiliary phase, were not included in this study. VEGF expression precedes angiogenesis, but important vascular changes may occur later than impaired hepatocyte function in early-stage HCC [32]. Compared with the arterial phase, the portal venous phase and hepatobiliary phase can reflect the functional changes in hepatocytes earlier, so we can find more information related to the function of hepatocytes from these characteristics to better predict the expression of VEGF than we can from the arterial phase [32].

The limitations of this study are as follows. First, the sample size of this study was limited, and external validation was lacking. Second, although the manual segmentation method has greater accuracy than semiautomatic segmentation and automatic segmentation [14, 15], it is still possible that inaccurate segmentation was caused by unclear boundaries in some images. Finally, selection bias may be present in a retrospective study.

## Conclusions

In summary, the radiomic model based on multisequence MR images has the potential to predict VEGF expression in HCC patients, and the combined model showed the best performance. These findings may be beneficial for choosing optimal and individualized treatment strategies for HCC patients.

## Data Availability

The data generated and analyzed during this study are available from the corresponding author on reasonable request.
